# Clinical Significance and Immune Landscape of a Pyroptosis-Derived LncRNA Signature for Glioblastoma

**DOI:** 10.3389/fcell.2022.805291

**Published:** 2022-02-10

**Authors:** Zhe Xing, Zaoqu Liu, Xudong Fu, Shaolong Zhou, Long Liu, Qin Dang, Chunguang Guo, Xiaoyong Ge, Taoyuan Lu, Youyang Zheng, Lirui Dai, Xinwei Han, Xinjun Wang

**Affiliations:** ^1^ Department of Neurosurgery, The Fifth Affiliated Hospital of Zhengzhou University, Zhengzhou, China; ^2^ Henan International Joint Laboratory of Glioma Metabolism and Microenvironment Research, Zhengzhou, China; ^3^ Department of Interventional Radiology, The First Affiliated Hospital of Zhengzhou University, Zhengzhou, China; ^4^ Department of Hepatobiliary and Pancreatic Surgery, The First Affiliated Hospital of Zhengzhou University, Zhengzhou, China; ^5^ Department of Colorectal Surgery, The First Affiliated Hospital of Zhengzhou University, Zhengzhou, China; ^6^ Department of Endovascular Surgery, The First Affiliated Hospital of Zhengzhou University, Zhengzhou, China; ^7^ Department of Cerebrovascular Disease, Zhengzhou University People’s Hospital, Zhengzhou, China; ^8^ Department of Cardiology, The First Affiliated Hospital of Zhengzhou University, Zhengzhou, China

**Keywords:** pyroptosis, long non-coding RNA, glioblastoma (GBM), prognostic signature, immune landscape, immunotherapy, chemotherapy

## Abstract

**Introduction:** Pyroptosis was recently implicated in the initiation and progression of tumors, including glioblastoma (GBM). This study aimed to explore the clinical significance of pyroptosis-related lncRNAs (PRLs) in GBM.

**Methods:** Three independent cohorts were retrieved from the TCGA and CGGA databases. The consensus clustering and weighted gene coexpression network analysis (WGCNA) were applied to identify PRLs. The LASSO algorithm was employed to develop and validate a pyroptosis-related lncRNA signature (PRLS) in three independent cohorts. The molecular characteristics, clinical significances, tumor microenvironment, immune checkpoints profiles, and benefits of chemotherapy and immunotherapy regarding to PRLS were also explored.

**Results:** In the WGCNA framework, a key module that highly correlated with pyroptosis was extracted for identifying PRLs. Univariate Cox analysis further revealed the associations between PRLs and overall survival. Based on the expression profiles of PRLs, the PRLS was initially developed in TCGA cohort (*n* = 143) and then validated in two CGGA cohorts (*n* = 374). Multivariate Cox analysis demonstrated that our PRLS model was an independent risk factor. More importantly, this signature displayed a stable and accurate performance in predicting prognosis at 1, 3, and 5 years, with all AUCs above 0.7. The decision curve analysis also indicated that our signature had promising clinical application. In addition, patients with high PRLS score suggested a more abundant immune infiltration, higher expression of immune checkpoint genes, and better response to immunotherapy but worse to chemotherapy.

**Conclusion:** A novel pyroptosis-related lncRNA signature with a robust performance was constructed and validated in multiple cohorts. This signature provided new perspectives for clinical management and precise treatments of GBM.

## Introduction

Glioblastoma (GBM) is the most commonly occurring type of glioma, according to the 2016 World Health Organization (WHO) classification, and is also the most lethal primary brain tumor worldwide, which is closely related to significant morbidity and mortality in adults, with a 5-year overall survival (OS) rate of 5% (Roos et al., 2017; Wesseling and Capper, 2018; Oronsky et al., 2020; Majc et al., 2021). Despite the application of the optimum therapeutic options, including surgical resection, radiotherapy, chemotherapy as well as tumor-treating field treatment (TTF), the OS remains poor (Stupp et al., 2017; Delgado-Martin and Medina, 2020; Tan et al., 2020).

Pyroptosis, a novel fashion of programmed cell death, referred to as cellular inflammatory necrosis (Kovacs and Miao, 2017), is triggered by inflammasomes and mainly executed by the cleavage of gasdermin proteins, such as gasdermin D (GSDMD) and gasdermin E (GSDME), which can be cleaved by caspase-1 and caspase-3, respectively, to spark off pyroptosis (Shi et al., 2015; Ding et al., 2016). Pyroptosis participates in the development of multiple tumors, including glioma. Upregulation of transcription factor p53 inhibits tumor growth through prompting pyroptosis in non-small-cell lung cancer (Zhang et al., 2019). Galangin can exert antitumor effects by inducing apoptosis, pyroptosis, and protective autophagy in GBM cells (Kong et al., 2019). Therefore, the impacts of pyroptosis on glioma cannot be ignored.

Long noncoding RNAs (lncRNAs), a sort of noncoding RNA longer than 200 nucleotides, are involved in a wide range of biological processes, such as cell death, growth, differentiation, posttranscriptional regulation, chromatin modification, inflammatory pathology, epigenetic regulation, and subcellular transport (Rynkeviciene et al., 2018). Recently, an increasing number of studies have shown that lncRNAs play important roles in the pyroptosis progress, by acting directly or indirectly on the pyroptosis signaling, to exert effects on a variety of diseases, including tumors (Xie et al., 2019; He et al., 2020; Wan et al., 2020; Xu et al., 2020; Wang et al., 2021). However, the roles of pyroptosis-related lncRNAs (PRLs) in GBM have never been reported.

In the present study, we identified and validated a novel pyroptosis-related lncRNA signature (PRLS) in three independent datasets. The molecular characteristics, clinical significances, tumor microenvironment, immune checkpoints profiles, and benefits of chemotherapy and immunotherapy regarding PRLS were also explored. The PRLS demonstrated the outstanding performances in prognosis prediction, and more importantly, PRLS also has implications for the immunotherapy and chemotherapy of different risk groups. In summary, we believe that the PRLS contributes to furnishing extra evidence for risk stratification and treatment guidance for GBM patients.

## Methods and materials

### Patient data collection and acquisition of long noncoding RNAs

The RNA-sequencing (RNA-seq) data with relevant clinical information of GBM patients were downloaded using UCSC Xena from the Cancer Genome Atlas (TCGA, https://portal.gdc.cancer.gov/). Two validation datasets were obtained from the Chinese Glioma Genome Atlas (CGGA, http://www.cgga.org.cn) database also including both the transcriptome data and the clinical characteristics. The clinical baseline data are summarized in the Supplementary Material: Supplementary Table S1.

A total of 51 pyroptosis-related genes (PRGs) were acquired from the REACTOME_PYROPTOSIS gene set in the Molecular Signatures Database (MSigDB) and prior published papers (Man and Kanneganti, 2015; Wang and Yin, 2017; Karki and Kanneganti, 2019; Xia et al., 2019), which are presented in Supplementary Table S2. Based on the annotation of the Genome Reference Consortium Human Build 38 (GRCh38), we extracted the expression matrix of 15,229 lncRNAs in the TCGA dataset (named TCGA) and 4,311 and 4,356 lncRNAs in the two CGGA datasets (named c325, c693), respectively.

### Consensus clustering

Based on the expression profiles of the 51 PRGs, the consensus clustering was performed to decipher heterogeneous subtypes in the TCGA dataset. This process was implemented *via* the “ConsensusClusterPlus” R package with the parameters of 500 iterations, resample rate of 0.8. The clustering heatmaps, empirical cumulative distribution function (CDF), and proportion of ambiguous clustering (PAC) analysis were illustrated based on k-value (2–9). We further performed the principal component analysis (PCA) to compare the differences between different groups based on the clustering results.

**Construction of weighted gene coexpression networks and identification of pyroptosis-related long noncoding RNAs in glioblastoma**

Gene coexpression network analysis was specifically performed on tumor tissues using the “WGCNA” R package. First, we selected the top 5,000 genes with median absolute deviation (MAD), and the samples with outlier were removed using the hclust algorithm. The minimum number of module genes was set at 30. The cutreeDynamic function was employed for tree pruning of the gene hierarchical clustering dendrograms generating coexpression modules and correlated modules (r >0.75) were merged. The disparity of the module Eigengenes (ME) was calculated using the module Eigengenes function. Correlation between Eigengenes values with the pyroptosis-related subtypes was evaluated by Pearson’s correlation coefficient. The module with the highest correlation coefficient was extracted for further investigation.

### Development and validation of pyroptosis-related long noncoding RNAs

For the genes in the identified module, univariate Cox regression was performed to determine the associations between genes and OS in the TCGA cohort. Then the LASSO Cox regression algorithm was applied to identify the key lncRNAs and construct a prediction model. The lncRNAs with nonzero coefficients were defined as the key lncRNAs. Based on these key lncRNAs, the prognostic risk score was calculated for each patient predicated upon the formula shown below:

$$Risk\;score=\sum_{i=1}^{n}Coef_{i}\times Expr_{i}$$

where *Coef*_*i*_ was defined as the coefficient of lncRNAs 1) and *Expr*_*i*_ represented the expression level. Patients were classified into high- and low-risk groups according to the median value. The Kaplan–Meier (KM) survival analysis and log-rank test were conducted to estimate survival difference between two groups using the “survminer” R packages. Time-dependent receiver operating characteristic (ROC) curves were profiled to examine the predictive accuracy of this model using the “survivalROC” R package, and the area under the curve (AUC) values demonstrated distinction. The decision curve analysis (DCA) was performed to evaluate the intended clinical effectiveness of this model. Subsequently, this PRLS model was further validated in two CGGA cohorts (c325 and c693).

### Gene set enrichment analysis

Gene set enrichment analysis (GSEA) was conducted to reveal the significantly enriched biological processes and potential molecular mechanisms regarding PRLS using the hallmark gene sets (h.all.v7.4. symbols). Hallmark summarizes and represents specific well-defined biological states or processes and demonstrates coherent expression (Liberzon et al., 2015). These gene sets were extensively utilized in cancer-related studies (Liu et al., 2021e; Liu et al., 2021f). The gene terms with |normalized enrichment score (NES)| >1 and false discovery rate (FDR) <0.01 were considered to be statistically significant.

### Cell infiltration

Five algorithms, including ESTIMATE, CIBERSORT, xCell, ssGSEA, and MCPcounter, were applied to evaluate the immune infiltration patterns based on the transcriptome expression in the TCGA dataset. Correlations between the PRLS and the immune cell infiltration were further explored.

### Evaluation of immune checkpoint profiles

The relationship of a total of 27 immune checkpoints with PRLS, including B7-CD28 family (PD-1, PD-L1, PD-L2, CTLA4, CD276, HHLA2, ICOS, ICOSLG, TMIGD2, and VTCN1), the TNF superfamily (BTLA, CD27, CD40, CD40LG, CD70, TNFRSF18, TNFRSF4, TNFRSF9, and TNFSF14), and several other molecules (ENTPD1, FGL1, HAVCR2, IDO1, LAG3, NCR3, NT5E, and SIGLEC15) (Liu et al., 2021c) were furthered explored.

### Immunotherapy assessment

The Tumor Immune Dysfunction and Exclusion (TIDE) and T-cell inflammatory signature (TIS) methods were applied to predict the immunotherapeutic response to immune checkpoint blockade (ICB) for each patient. The TIDE algorithm is a kind of computational approach, with modules of two individual mechanisms of tumor immune evasion, including T-cell dysfunction and T-cell exclusion in tumors (Jiang et al., 2018). The TIS, proposed by Ayers et al. could predict the putative efficacy of PD-1 inhibitors (Ayers et al., 2017). We then carried out the Subclass Mapping (SubMap) method, which employs the GSEA algorithm to evaluate the similarity of expression profiles between risk groups and patients with different responses to immunotherapy.

### Estimation of the sensitivity of chemotherapeutic agents

We further applied the “pRRophetic” R package to estimate the chemotherapeutic response by predicting the half-maximal inhibitory concentration (IC50) of 138 agents between two groups (Liu et al., 2021b; Liu et al., 2021d). The “pRRophetic” R package utilized a ridge regression model to use expression data as predictors and output as drug sensitivity values (of the drug of interest) (Geeleher et al., 2014). Higher IC50 indicated higher drug sensitivity.

### Statistics

All data processing, statistical analysis, and plotting were conducted in the R 4.0.5 software. Fisher’s exact test or Pearson’s Chi-squared test was applied to compare categorical variables. The Wilcoxon rank-sum test or t-test was utilized in continuous variables between the two groups. All *p*-values were two-sided, with *p*-value <0.05 deemed as statistically significant.

## Results

### Identification of prognostic pyroptosis-related lncRNAs in glioblastoma patients

**FIGURE 1:**
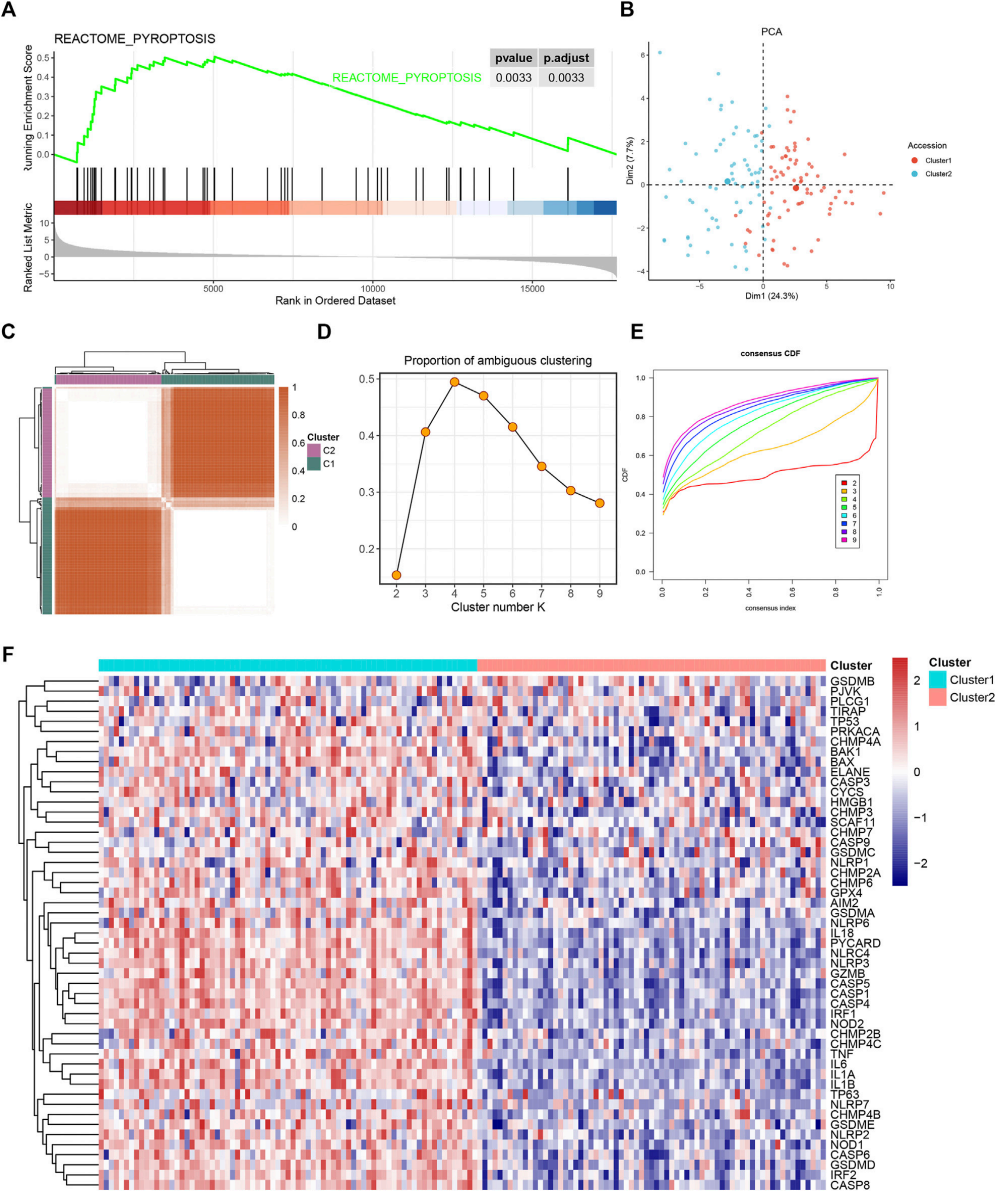
The gene set enrichment analysis (GSEA) and consensus clustering of pyroptosis-related gene set. **(A)** Gene set enrichment analysis showed pyroptosis-related signatures significantly enriched in glioblastoma (GBM) patients. **(B)** Two-dimensional principle component plot by gene profile of 51 pyroptosis-related genes (PRGs). Each point represents a single sample, with different colors indicating the different clusters. **(C)** The clustering heatmap of GBM samples when k = 2. **(D)** The proportion of ambiguous clustering (PAC) score; a low value of PAC implies a flat middle segment, allowing conjecture of the optimal k (k = 2) by the lowest PAC. **(E)** The cumulative distribution functions of clustering heatmaps for each k (indicated by colors). **(F)** The pyroptosis cluster of GBM patients in The Cancer Genome Atlas (TCGA) cohort.

**FIGURE 2:**
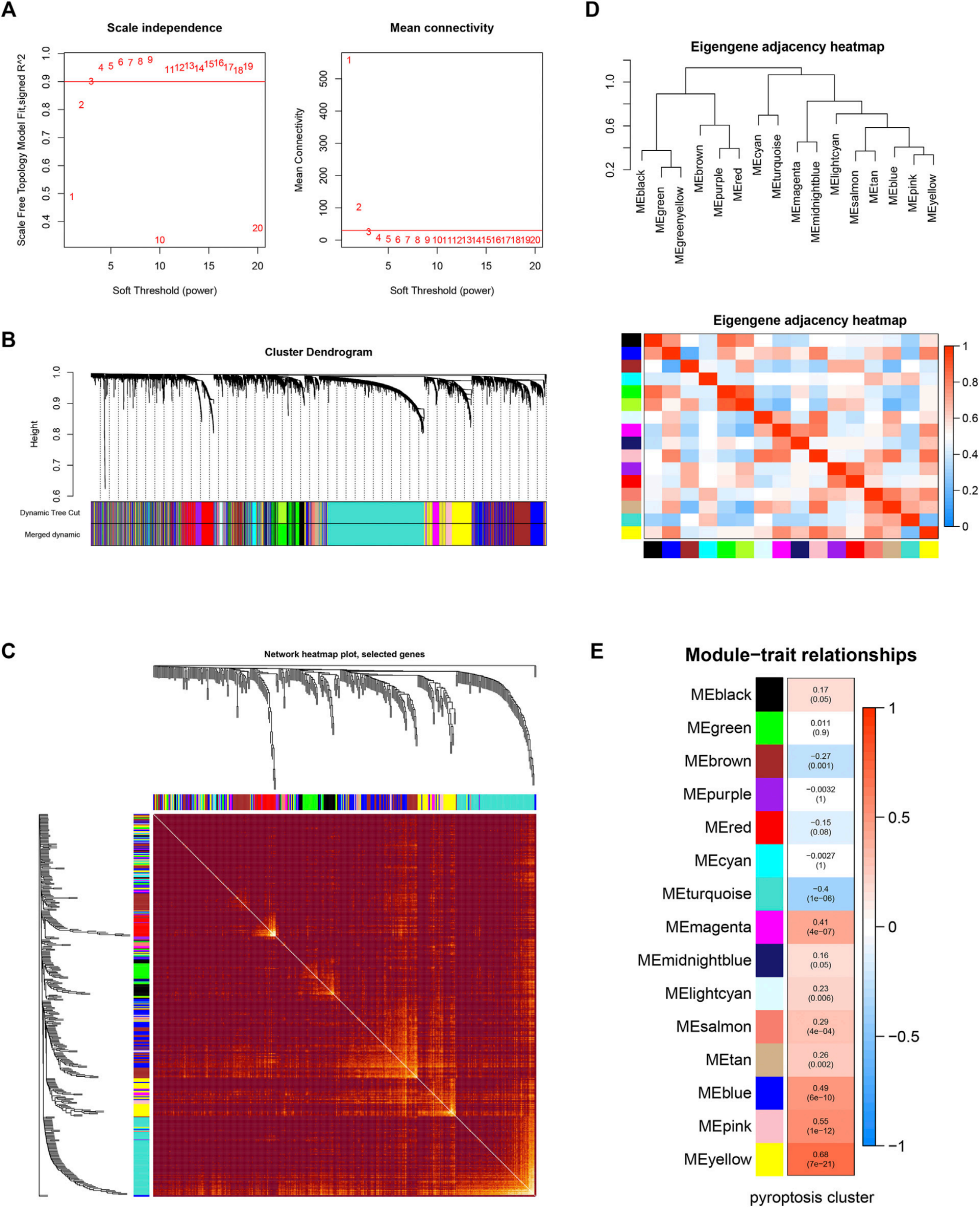
Weighted gene coexpression network analysis. **(A)** Analysis of network topology for various soft-thresholding powers. The left panel shows the scale-free fit index (*y*-axis) as a function of the soft-thresholding power (*x*-axis). The right panel displays the mean connectivity (degree, *y*-axis) as a function of the soft-thresholding power (*x*-axis). **(B)** Clustering dendrogram of genes, with dissimilarity based on topological overlap, together with assigned module colors. **(C)** Visualizing the gene network using a heatmap plot. The heatmap depicts the Topological Overlap Matrix (TOM) among all genes in the analysis. Light color represents low overlap and the progressively darker red color represents a higher overlap. Blocks of darker colors along the diagonal are the modules. **(D)** Visualization of the eigengene network representing the relationships among the modules and the clinical trait weight. **(E)** Module-trait associations: Each row corresponds to a module eigengene and the column to the pyroptosis cluster. Each cell contains the corresponding correlation and *p*-value.

**FIGURE 3:**
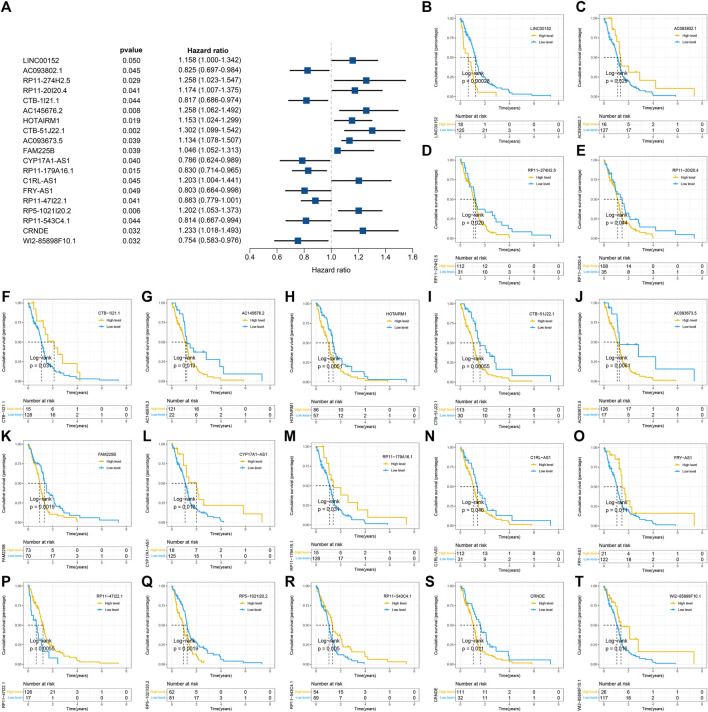
Identification and of prognostic pyroptosis-related long noncoding RNAs (lncRNAs) and further Kaplan–Meier (KM) analysis. **(A)** Univariate Cox regression was utilized to identify 19 prognostic pyroptosis-related lncRNAs, and the corresponding *p*-values and hazard ratio values were also exhibited. **(B–T)** KM curves were illustrated to exhibit the relationship between overall survival (OS) and the expression levels of these 19 pyroptosis-related lncRNAs (PRLs) based on the optimal cutoff points.

The workflow of our study is shown in Supplementary Figure S1. The GSEA was performed on 51 PRGs to assess whether pyroptosis was significantly associated with GBM in our study. The results demonstrated that the pyroptosis pathway of GBM is dysregulated compared with normal samples (Figure 1A). Subsequently, the clustering analysis classified the patients into different clusters, based on the expression levels of the PRGs. Eventually, k = 2 was determined as the optimal clustering number according to the clustering heatmaps, PAC analysis, and CDF curves (Figures 1C–E). GBM patients in TCGA cohort were clustered into two clusters. The PCA results demonstrated the spatial distribution of gene profiles, and the two clusters were distributed into distinct directions indicating that pyroptosis genes can distinguish GBM patients (Figure 1B). The heatmap demonstrated higher expression levels of PRGs in cluster 1 versus cluster 2 (Figure 1F). As expected, most of the PRGs were overexpressed in cluster 1 (Supplementary Figure S2). In recognizing the lncRNAs associated with pyroptosis cluster, the “WGCNA” R package was employed to construct the weighted coexpression network. The soft power of β = 3 (scale-free *R* ^2^ = 0.95) was chosen for the soft thresholding to acquire coexpressed gene modules (Figure 2A). Ultimately, we obtained a total of 15 modules for subsequent analysis, the heatmap graph of topological overlap matrix (TOM), and the relationships among the modules are illustrated in Figure 2. The yellow module composed of 616 lncRNAs showed the highest gene significance with pyroptosis cluster and, hence, was selected for further analysis (Figure 2E and Supplementary Figure S3). Based on the selected module genes, we obtained 19 prognostic PRLs shared by three datasets. KM analysis was exploited to investigate the relationship between the expression levels of these 19 PRLs and OS. Consistent with the results of univariate Cox regression, 11 of the PRLs (LINC00152, RP11-274H2.5, RP11-20I20.4, AC145676.2, HOTAIRM1, CTB-51J22.1, AC093673.5, FAM225B, C1RL-AS1, RP5-1021I20.2, and CRNDE) were risky factors, while the remaining 8 were protective factors, including AC093802.1, CTB-1I21.1, CYP17A1-AS1, RP11-179A16.1, FRY-AS1, RP11-47I22.1, RP11-543C4.1, and WI2-85898F10.1 (Figure 3). Furthermore, the coexpression relationships between the 51 PRGs and the 19 lncRNAs were investigated (Supplementary Figure S4).

### Construction and verification of the pyroptosis-related long noncoding RNAs

**FIGURE 4:**
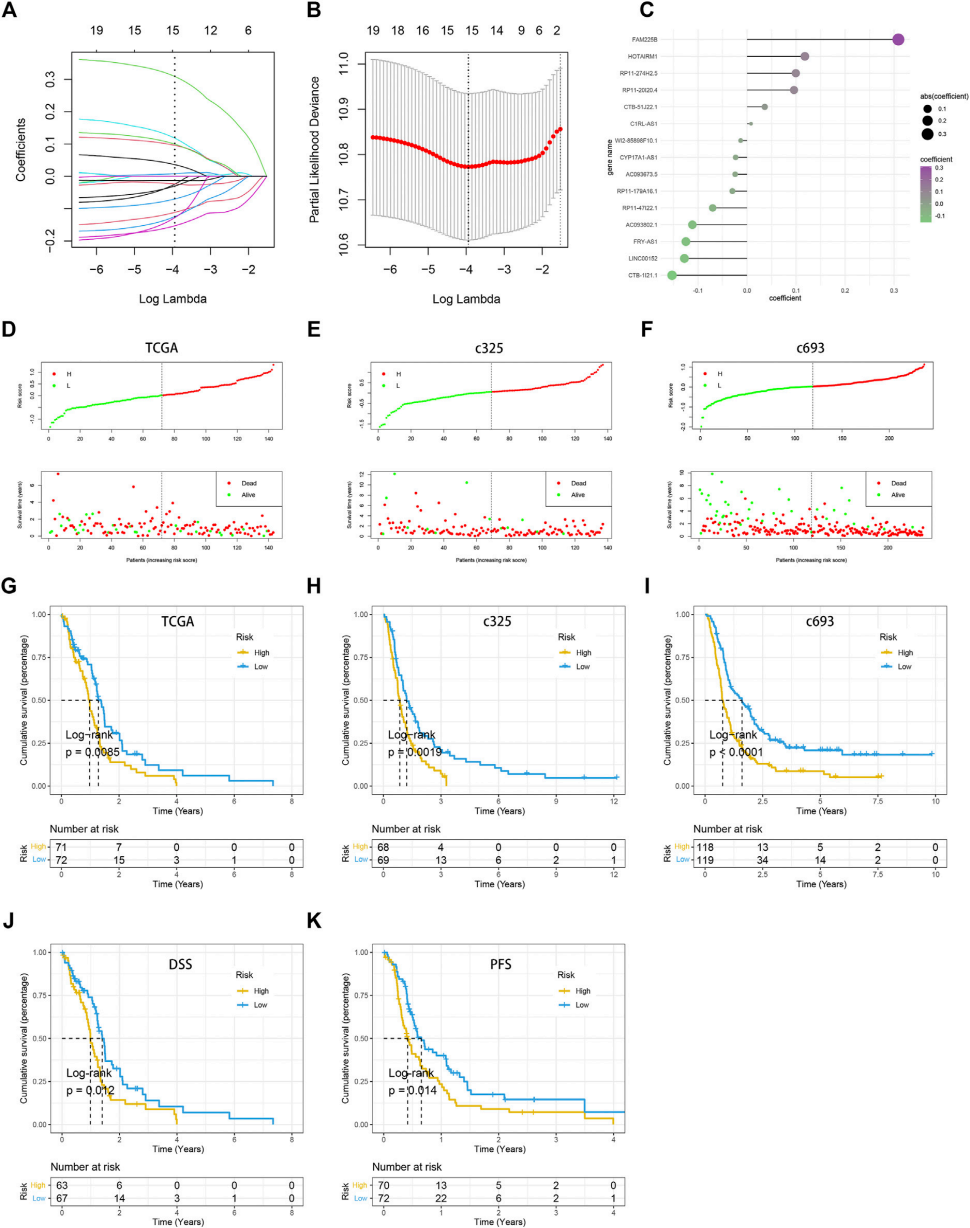
Construction of the pyroptosis-related lncRNA signature (PRLS). **(A)** LASSO coefficient profiles of the candidate PRLs for PRLS construction. **(B)** Ten-time cross-validations to tune the parameter selection in the LASSO model. The two dotted vertical lines are drawn at the optimal values by minimum criteria (left) and 1-SE criteria (right). **(C)** LASSO coefficient profiles of the candidate genes for RAIS construction. **(D–F)** The scattergrams of the risk score (up) and survival status (down) of each patient in the TCGA, c325, c693 cohorts, respectively. In the upper parts of the scattergrams, the red and green dots represent high-risk (“H”) and low-risk groups (“L”), respectively, and in the lower part of the scattergrams, death and survival, respectively. **(G–I)** Kaplan–Meier overall survival (OS) analysis of the high-risk and low-risk groups based on the PRLS and median risk scores in the TCGA, c325, c693 cohorts, respectively. **(J)** Kaplan–Meier curve of disease-specific survival (DSS) in the TCGA cohort. **(K)** Kaplan–Meier curve of progression free survival (PFS) in the TCGA cohort.

**FIGURE 5:**
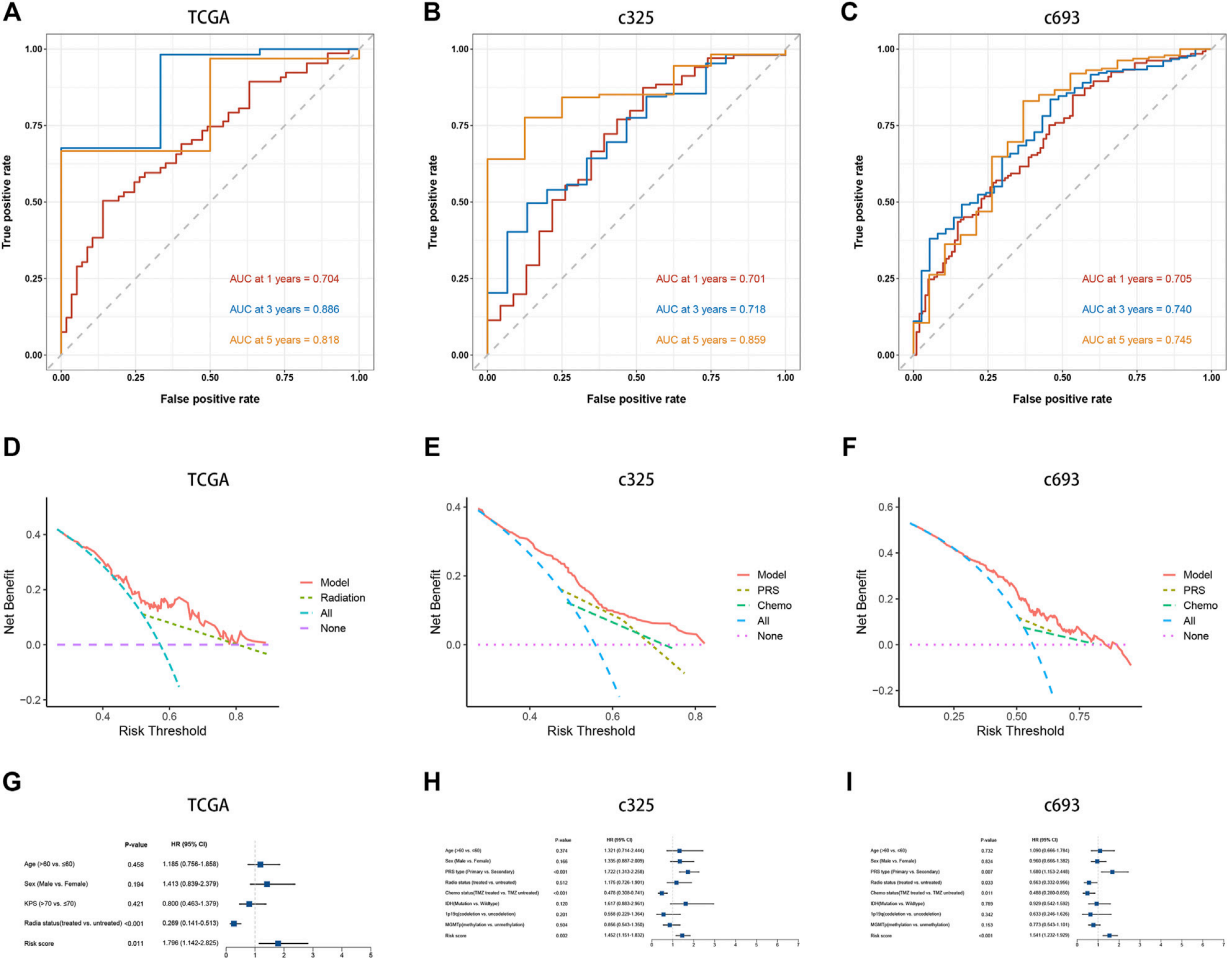
Prediction performance and independence of the PRLS. **(A–C)** Time-dependent receiver operating characteristic curve (ROC) analysis for predicting overall survival (OS) at 1-, 3-, and 5-years **(D–F)** Decision curve analysis (DCA) curves to evaluate the clinical utility of different decision strategies, and the red line represented the PRLS. **(G–H)** Multivariate Cox regression analysis of the risk score in three cohorts.

The LASSO Cox analysis was performed to generate a pyroptosis-related lncRNA signature of 15 PRLs (Figures 4A, B), including FAM225B, HOTAIRM1, RP11-274H2.5, RP11-20I20.4, CTB-51J22.1, C1RL-AS1, WI2-85898F10.1, CYP17A1-AS1, AC093673.5, RP11-179A16.1, RP11-47I22.1, AC093802.1, FRY-AS1, LINC00152, and CTB-1I21.1, of which regression coefficients are exhibited in Figure 4C. A risk score for each patient was calculated based on the expression and coefficients of PRLs, then the GBM patients were divided into two subgroups (low- and high-risk groups) using the median value in TCGA cohort. The KM survival curves show that GBM patients in the high-risk group had significantly shorter OS compared with the low-risk group (Figure 4G). The scattergrams of the risk score and survival status revealed shorter OS time and more dismal events with increasing risk score (Figure 4D). The promising prediction ability of PRLS was corroborated by the ROC curves for 1-, 3-, and 5-year OS rates (AUC = 0.704, 0.886, and 0.818, respectively; Figures 5A, D). Decision curves showed the highest net benefit for the PRLS (“Model”) compared with default strategies (“All” and “None”) and clinical traits with prognostic significance (“Radiation” represented radiotherapy). Multivariate Cox regression analysis demonstrated that the PRLS was an independent prognostic factor even including available clinical variables (Figure 5G). Additionally, the disease-specific survival (DSS) and progression-free survival (PFS) regarding PRLS were further scrutinized. Patients in the high-risk group showed longer DSS as well as PFS (Figures 4J, K).

### Assessment of the prognostic pyroptosis signature in external test cohorts

In two CGGA cohorts, c325 and c693, as we did in the TCGA database, the GBM patients were classified into high- and low-risk groups based on cutoff values of median risk scores as well (Figures 4H, I). The signature in both validation datasets showed favorable predictive performances for OS rates of 1-, 3-, and 5-years (AUC = 0.701, 0.718, and 0.859 in c325 and AUC = 0.705, 0.740, and 0.745 in c693, respectively Figures 5B, C). The DCA also revealed the highest predictive value of clinical prognosis for the PRLS (“Model”) compared with the default strategies (“All” and “None”) and clinical variables with prognostic significance (“PRS” represented primary/secondary status; “Chemo” represented chemotherapy) (Figures 5E, F). In line with the TCGA training cohort, our PRLS model could independently predict the prognosis of GBM patients in two external test cohorts (Figures 5H, I).

### Immune landscape of pyroptosis-related long noncoding RNAs

**FIGURE 6:**
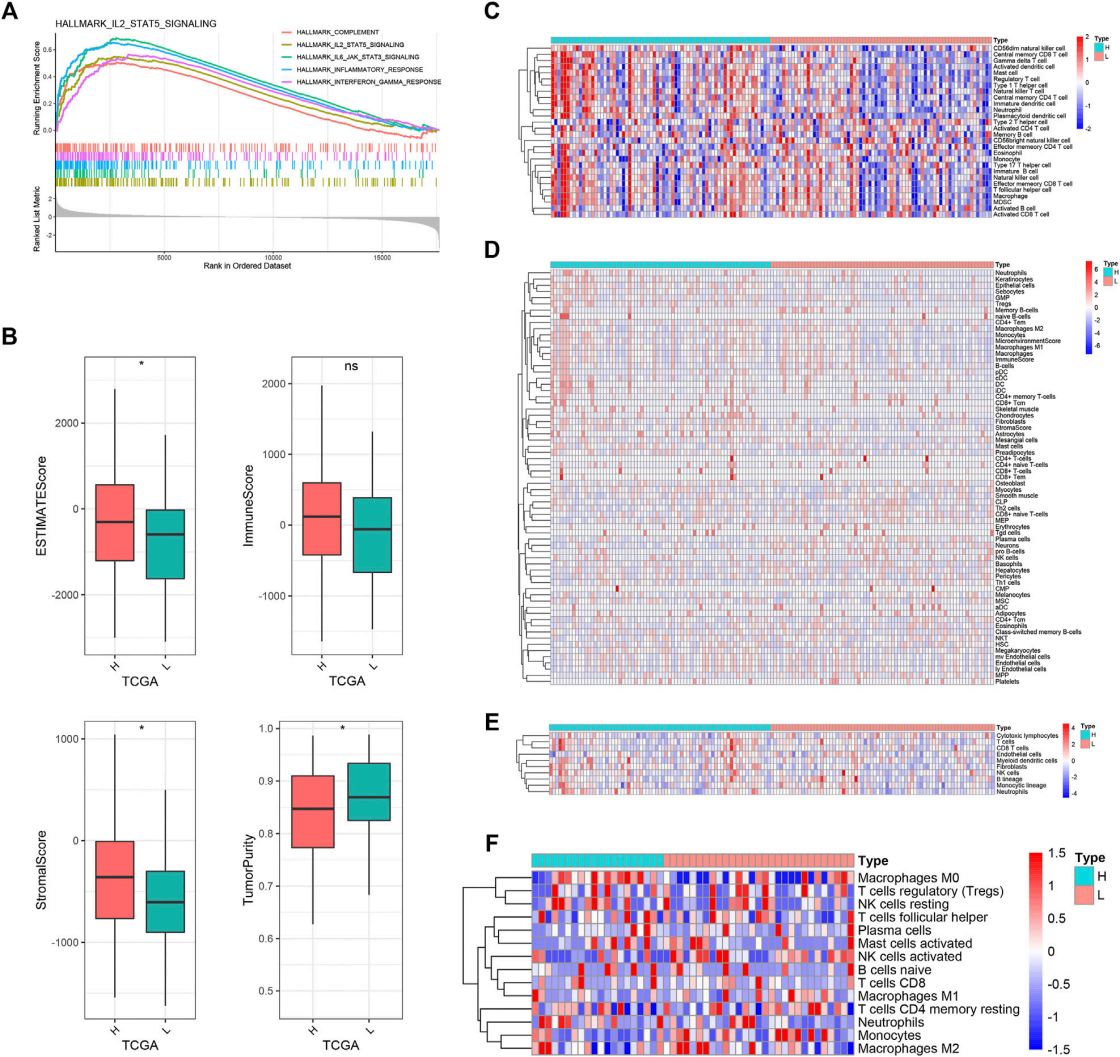
Biological functions and immune landscape regarding PRLS. **(A)** The enriched gene sets in HALLMARK collection by the high-risk groups. Each line representing one particular gene set with unique color, and upregulated genes located in the left approaching the origin of the coordinates; by contrast the downregulated lay on the right of *x*-axis. Only gene sets with normalized enrichment score |NES| >2 and false discovery rate (FDR) <0.01 were considered significant. Only several leading gene sets were displayed in the plot. **(B)** The distribution difference of ESTIMATE, immune, stromal, and tumor purity enrichment score between the high-risk and low-risk groups. ^ns^p > 0.05, *p < 0.05. **(C–F)** Heatmaps of immune cells infiltration in the high-risk and low-risk groups based on ssGSEA, xCell, MCPcounter, and CIBERSORT algorithms, respectively.

The GSEA was carried out to explore the potential biological processes and mechanisms connected with different risk groups of GBM. The results manifested that the high-risk group enriched multiple immune-related hallmarks, including IL2_STAT5 signaling (NES = 2.24, FDR < 0.01), IL6_JAK_STAT3 signaling (NES = 2.46, FDR < 0.01), inflammatory response (NES = 2.66, FDR < 0.01), interferon_gamma response (NES = 2.31, FDR < 0.01), and complement (NES = 2.07, FDR < 0.01) (Figure 6A). Pathways closely related to the low-risk group showed limited significance with immunity, such as spermatogenesis (NES = −1.89, FDR < 0.01), pancreas_beta_cells (NES = −1.80, FDR < 0.01), KRAS_signaling (NES = −1.76, FDR < 0.01), E2F_targets (NES = −1.64, FDR < 0.01), and oxidative_phosphorylation (NES = −1.59, FDR < 0.01) (Supplementary Figure S5A).

Subsequently, we further investigated the immune landscape of PRLS. The ESTIMATE software was employed to deduce the proportion of stromal and immune fractions. The high-risk group showed the superior overall, stromal, and immune scores and lower tumor purity than the low-risk group in the TCGA dataset (Figure 6B). We then performed CIBERSORT, xCell, ssGSEA, and MCPcounter methods to further evaluate the proportion of immune cells. Overall, the infiltration of immune cells was more abundant in the high-risk group, and indicated more active immune status and immune response compared with the low-risk group (Figures 6C–F). The correlations between the PRLS and the immune cell infiltrations were further examined, some of which showed a higher level of immune infiltration in the high-risk group compared with the low-risk group, mainly comprising the T-cell family, such as central memory CD4 T cell, effector memory CD8 T cell, gamma delta T cell, type 1 T-helper cell, gamma delta T cell, natural killer T cell, neutrophil, CD56dim natural killer cell, plasmacytoid dendritic cell, etc. (Supplementary Figures S5B, C).

### Evaluation of the immune checkpoint profiles and the efficacy of immunotherapy

**FIGURE 7:**
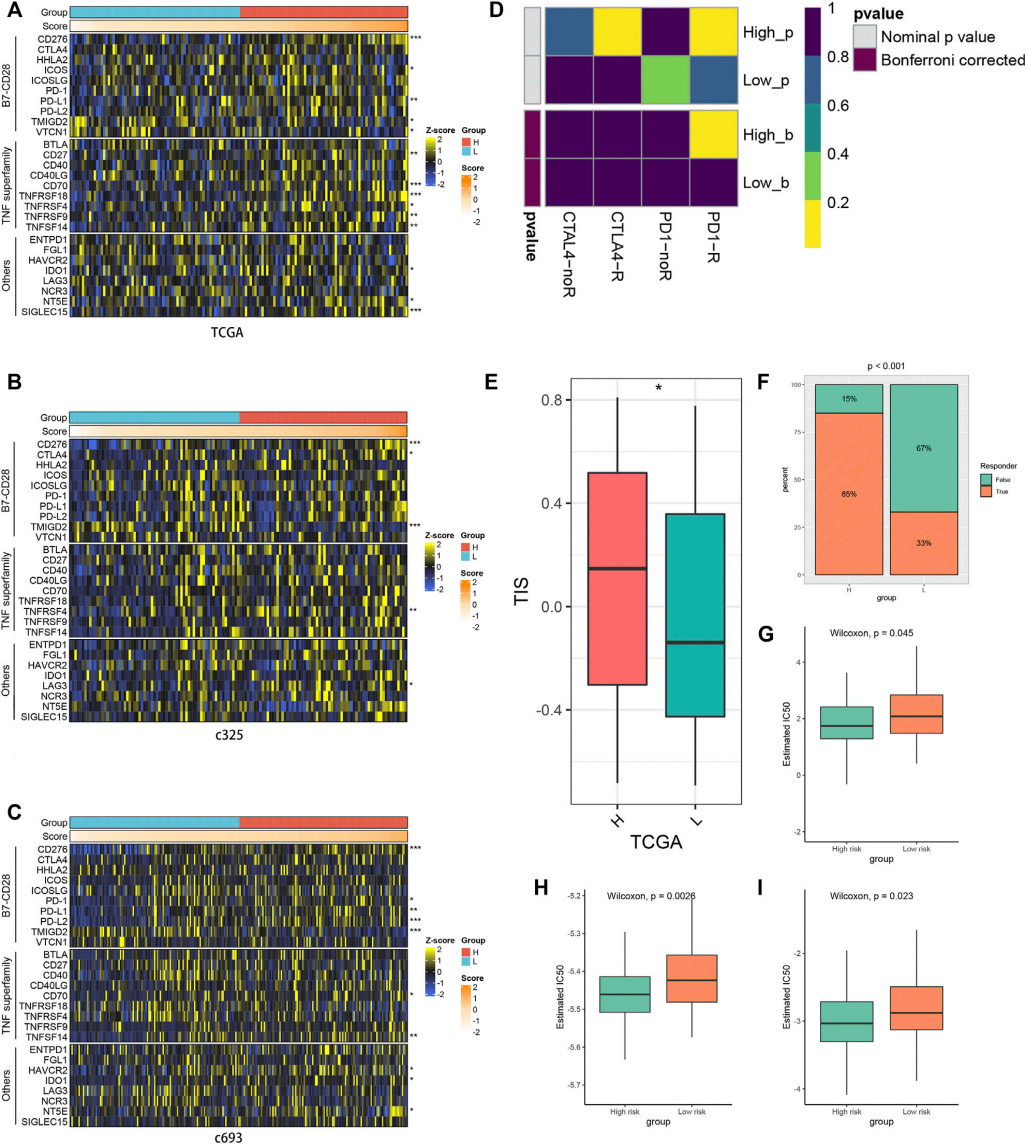
Evaluation of immune checkpoint profiles, immunotherapy, and chemotherapy between risk groups. **(A–C)** Three heatmaps of 27 immune checkpoints profiles in high-risk and low-risk groups. *p < 0.05, **p < 0.01, ***p < 0.001. **(D)** Submap analysis of the TCGA cohort and 47 previous melanoma patients with detailed immunotherapeutic information. **(E)** The distribution difference of T-cell inflammatory signature (TIS) score between the high- and low-risk groups. *p < 0.05. **(F)** Distribution of the immunotherapy response results predicted by the Tumor Immune Dysfunction and Exclusion (TIDE) algorithm among the high- and low-risk groups in the TCGA cohort. **(G–I)** Distribution of the estimated half-maximal inhibitory concentration (IC50) of bleomycin **(G)**, docetaxel **(H)**, and paclitaxel **(I)** between the high- and low-risk groups in TCGA cohort.

The connections between immune checkpoints and the PRLS in three cohorts are presented in Figures 7A–C. Overall, most of them were significantly upregulated in the high-risk group, and it was noteworthy that CD276 and TMIGD2 showed this trend in all three cohorts, which suggested being potential therapeutic targets. The TIDE web tool and the TIS algorithm were applied to infer the responses to ICB. The results demonstrated that patients in the high-risk group showed more responders and higher TIS score, which indicated that they were more inclined to derive considerable clinical benefit from ICB treatment. The SubMap algorithm was performed to explore the response to immunotherapy based on 47 previous melanoma patients with immunotherapeutic information; as expected, the high-risk group showed better immunotherapy sensitivity (Figures 7D–F).

### Other chemotherapeutic agents

Furthermore, we utilized the pRRophetic algorithm to predict the sensitivity to 138 drugs in the high- and low-risk groups. The results revealed 70 drugs with a significant variation of treatment sensitivity between the high- and low-risk groups (Supplementary Figure S6). Most of the discrepant drugs displayed enhanced responses in the low-risk group, and the majority of which were gene-targeting drugs, such as BMS-536924, an orally available GF-1R/IR inhibitor in glioma, which inhibits viability and migration of glioma cells and suppresses glioma tumor growth (Zhou, 2015). There were also some chemotherapy drugs. Bleomycin, a water-soluble glycopeptide antibiotic, induces single- and double-stranded DNA breaks and can be enhanced by photochemical internalization (PCI) in glioma cells (Mathews et al., 2012) and showed higher sensitivity in the low-risk group as well. For two other chemotherapy drugs, docetaxel and paclitaxel, the differences of treatment between the two groups were in keeping with bleomycin (Figures 7G–I). These results provided new perspectives for adjuvant treatments in GBM.

## Discussion

A total of 517 GBM patients from TCGA and CGGA datasets were involved in our study to probe into the prognostic significance of pyroptosis-related lncRNAs. Nineteen PRLs were recognized to have prognostic value in all three public databases (TCGA, c325, and c693), and 15 of them were included to construct a PRLS for predicting the OS of GBM patients by using LASSO Cox analysis. Based on the median risk score as the cutoff value, GBM patients were divided into the high- and low-risk subgroups, and the former had worse clinical outcomes. ROC curves and DCA demonstrated good predictive power of our model. Multivariate Cox regression analysis showed that PRLS was an independent risk factor for OS. Furthermore, we utilized several current acknowledged algorithms to reveal the immune characteristics between subgroups. In general, the high-risk group showed higher immune infiltration fraction and activity. The immunotherapy and chemotherapy responses were further explored regarding the PRLS. Patients in the high-risk group showed higher sensitivity to immunotherapy but less to chemotherapy. Overall, the PRLS we developed showed excellent performance in assessing the immune landscape and the prognosis prediction and treatment of GBM patients; thus, the specific application in clinical practice in the future is worth expecting.

Pyroptosis is a hotspot that has been increasingly studied in recent years, and many research have demonstrated that it is involved in the body’s inflammatory responses and closely related to the growth, development, and metastasis of various tumors (Xia et al., 2019; Fang et al., 2020; Ruan et al., 2020), but how it performs in an lncRNA-dependent manner during glioma progression remains unclear. Pyroptosis regulated a variety of biological processes in tumors mediated by lncRNAs. Downregulation of lncRNA-XIST suppressed the progression of non-small cell lung cancer (NSCLC) by triggering pyroptosis cell death mediated by miR-335/SOD2/ROS signal pathway (Liu et al., 2019). LncRNA ADAMTS9-AS2 activated pyroptosis cell death mediated by NLRP3 through sponging miR-223-3p to increase the sensitivity of cisplatin in gastric cancer (GC) to inhibit tumor development (Ren et al., 2020). Furthermore, lncRNA RP1-85F18.6 retrained the pyroptosis in colorectal cancer (CRC) by regulating the expression of ΔNp63 and promoted the proliferation and invasion of tumor cells. Having listed all the above, we believe that more attention should be paid to the roles of lncRNAs in the process of pyroptosis to determine potential prognostic markers and therapeutic targets of cancers.

We constructed a 15-PRL signature contributing to providing a novel tool of prognosis prediction, and some of them appeared in previous studies. FAM225B was found upregulated in recurrent GBMs (rGBMs), which was associated with the poor prognosis of rGBM patients (Li et al., 2020). High expression of HOTAIRM1 increased the evasion and migration of GBM cells (Xie et al., 2020). LINC00152 showed upregulated level in the mesenchymal subtype and isocitrate dehydrogenase1 wild type, and the expression of LINC00152 was increased with glioma grade (Wang et al., 2018). These genes mentioned above, which resulted in adverse outcomes with increased expression levels, also proved to be risk factors in the PRLS model. FRY-AS1 was demonstrated as a protective factor according to the research of Niu et al. (2020), which was consistent with the results of our study as well. Otherwise, some of the genes included in the PRLS have not been elucidated in glioma but in other tumors. A high expression level of C1RL-AS1 was shown to be associated with dismal prognosis of GC; similar results were presented in our study of GBM (Liu R. et al., 2021). There are also several genes that have never been reported in any tumor, which have significant prognostic value in our study, thus, needing further research.

Based on the PRLS, we aimed to interrogate and compare the characteristic differences in the immune microenvironment between tumor subgroups. GSEA demonstrated that the high-risk group is enriched in IL2-STAT5 signaling, IL6-JAK-STAT3 signaling, inflammatory response, interferon-gamma response, and complement in hallmark analysis, which suggested improved immunocompetence. The CIBERSORT, ssGSEA, xCell, and MCPcounter algorithms were applied to explore the immune cell infiltration in two groups, and some of them were found increased in the high-risk group, such as central memory CD4 T cell, central memory CD8 T cell, natural killer T cell, neutrophil, and so on. Moreover, we also detected enhanced levels of immune check points in the high-risk group. Tumor immune microenvironment, mostly consisting of stromal immune cells, plays vital roles in the proliferation, migration, and invasion of tumors and may be a crucial determinant of response to immune checkpoint blockade therapy (Mohme et al., 2020; Liu R. et al., 2021; Zhang et al., 2021). Unsurprisingly, the high-risk group showed higher sensitivity to immunotherapy, which indicated our model points out new directions and shows optimistic clinical application prospects in distinguishing patients suitable for immunotherapy.

To further explore the underlying role of our model in clinical decision making, we extensively interrogated the sensitivity of various drugs, with emphasis on gene-targeted drugs and potential chemotherapeutic drugs regarding PRLS. BMS-536924, a GF-1R/IR inhibitor that suppresses the growth of glioma, showed better responses in the low-risk group. Similarly, several kinds of chemotherapy drugs like bleomycin, docetaxel, and paclitaxel also showed a better propensity for benefit in the low-risk group. This may provide more opportunities for patients to receive effective treatment early and curb tumor progression to prolong survival.

The present study has some limitations as well. First, the clinical information of public datasets was very insufficient; hence, the potential connections between risk groups and important clinical characteristics may be disregarded. Second, our work focused on all types of glioblastomas without taking molecular subtypes, spatial heterogeneity, and so forth, into account. Notably, patients with IDH mutation exhibited better outcome compared with patients with IDH wild type (Molinaro et al., 2020). More detailed comparisons of diverse subtypes of GBM demand substantial clinical and sequencing data to secure progressive results. Third, several genes we submitted have not been particularly studied yet, thus, corroborative experiments deserve future research. Last, machine learning algorithms were implemented to explore the efficiency of different groups to immunotherapy and chemotherapy; nevertheless, further clinical validation is needed.

In summary, we developed and verified a novel pyroptosis-related lncRNA signature with remarkable ability and stability for survival prediction in GBM. Patients in the high PRLS group displayed enhanced immune activity and better efficacy of immunotherapy, while those in the low PRLS group tended to benefit more from chemotherapy. These results amplified the perception of PRLs in GBM and facilitated precise treatment and clinical management.

## Data Availability

The original contributions presented in the study are included in the article/Supplementary Material, further inquiries can be directed to the corresponding authors.

## Glossary

AUC: area under the curve
CDF: cumulative distribution function
CGGA: Chinese Glioma Genome Atlas
CRC: colorectal cancer
DCA: decision curve analysis
FDR: false discovery rate
GBM: glioblastoma
GC: gastric cancer
GRCh38: Genome Reference Consortium Human Build 38
GSDMD: gasdermin D
GSDME: gasdermin E
GSEA: Gene set enrichment analysis
IC50: half-maximal inhibitory concentration
ICB: immune checkpoint blockade
KM: Kaplan–Meier
lncRNA: Long non-coding RNA
MAD: median absolute deviation
ME: module Eigengene
MSigDB: Molecular Signatures Database
NES: normalized enrichment score
NSCLC: non-small cell lung cancer
OS: overall survival
PAC: proportion of ambiguous clustering
PCA: principal component analysis
PCI: photochemical internalization
PRG: pyroptosis-related genes
PRL: pyroptosis-related lncRNA
PRLS: pyroptosis-related lncRNA signature
rGBM: recurrent GBM
RNA-seq: RNA-sequencing
ROC curve: receiver operating characteristic curve
SubMap: Subclass Mapping
TCGA: the Cancer Genome Atlas
TIDE: Tumor Immune Dysfunction and Exclusion
TIS: T-cell inflammatory signature
TOM: topological overlap matrix
WGCNA: gene co-expression network analysis
WHO: World Health Organization
