# Supplementary figures and images for: Clinical Significance and Immune Landscape of a Pyroptosis-Derived LncRNA Signature for Glioblastoma
Source: Front Cell Dev Biol. 2022 Feb 10;10:805291. doi: 10.3389/fcell.2022.805291 (PMC8866949; doi:10.3389/fcell.2022.805291)

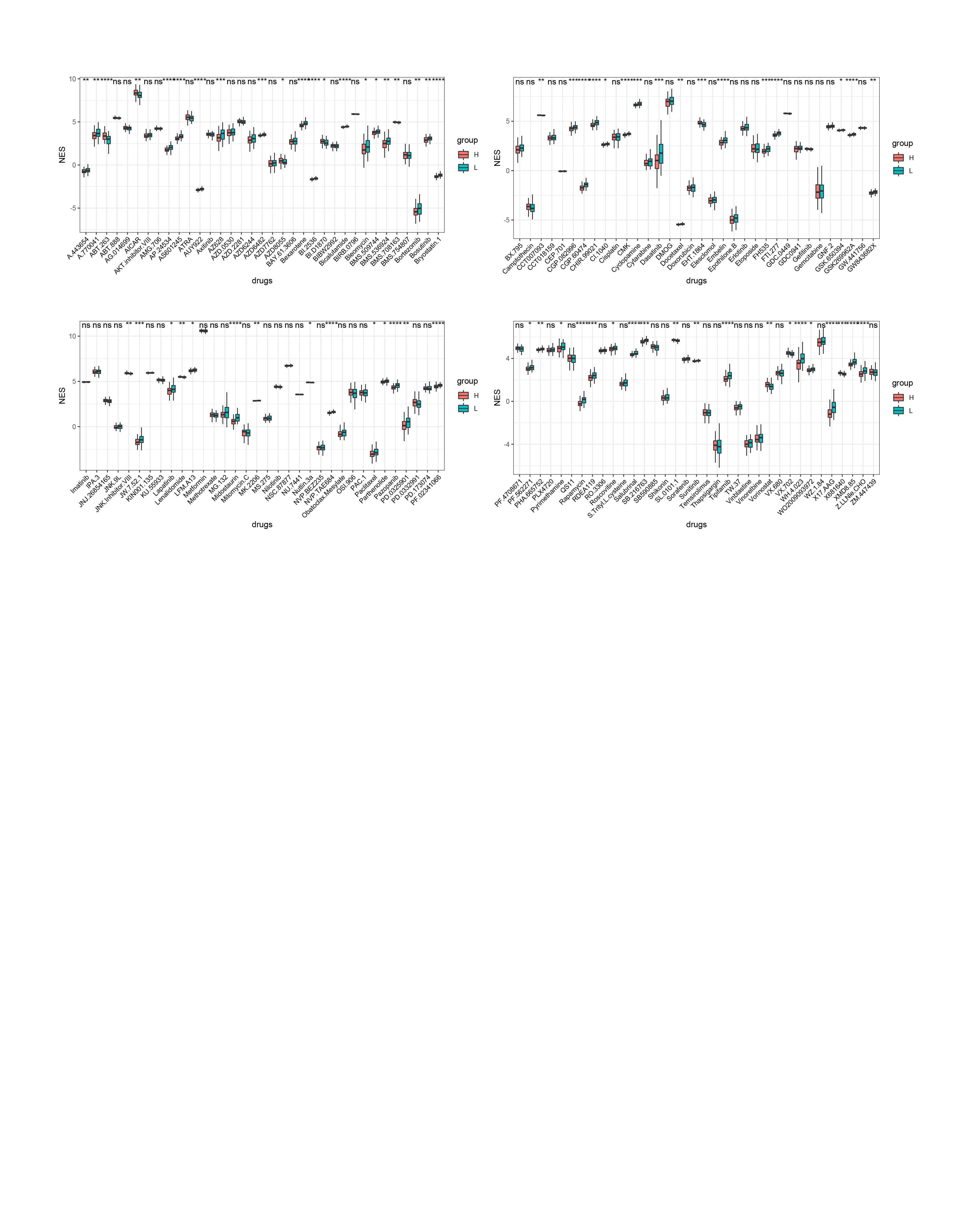

Supplement: Supplementary file 2 [file Image6.TIF]

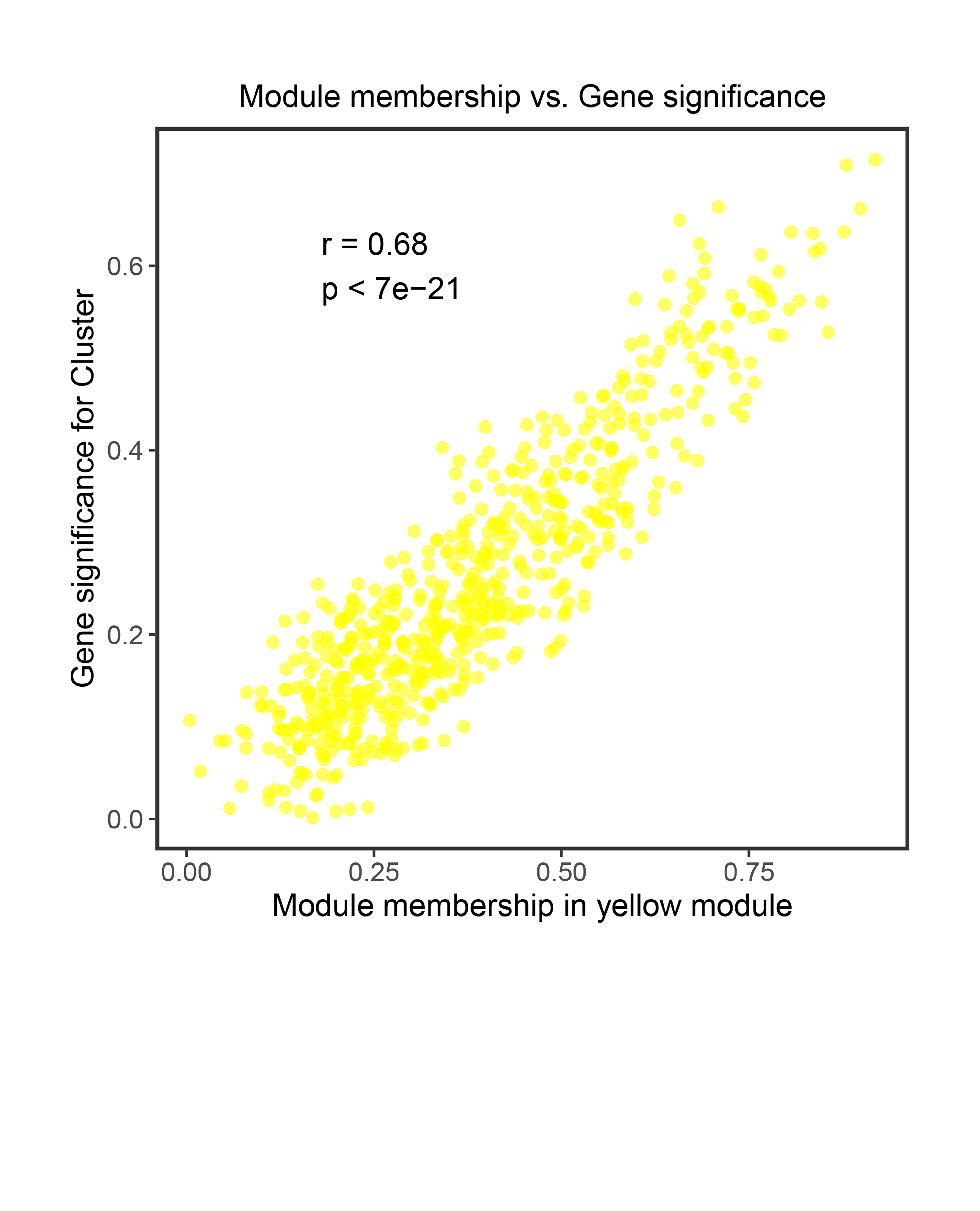

Supplement: Supplementary file 3 [file Image3.TIF]

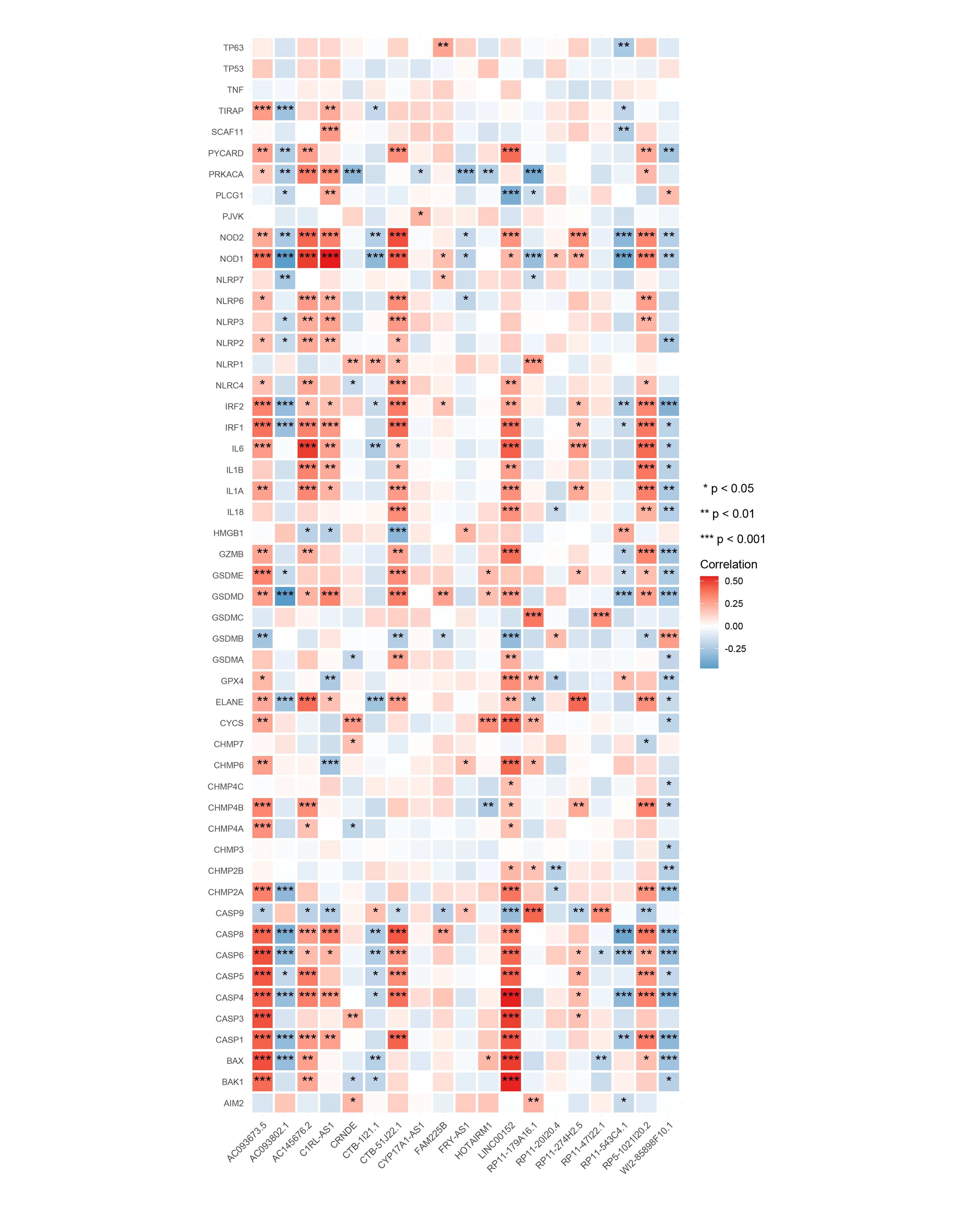

Supplement: Supplementary file 4 [file Image4.TIF]

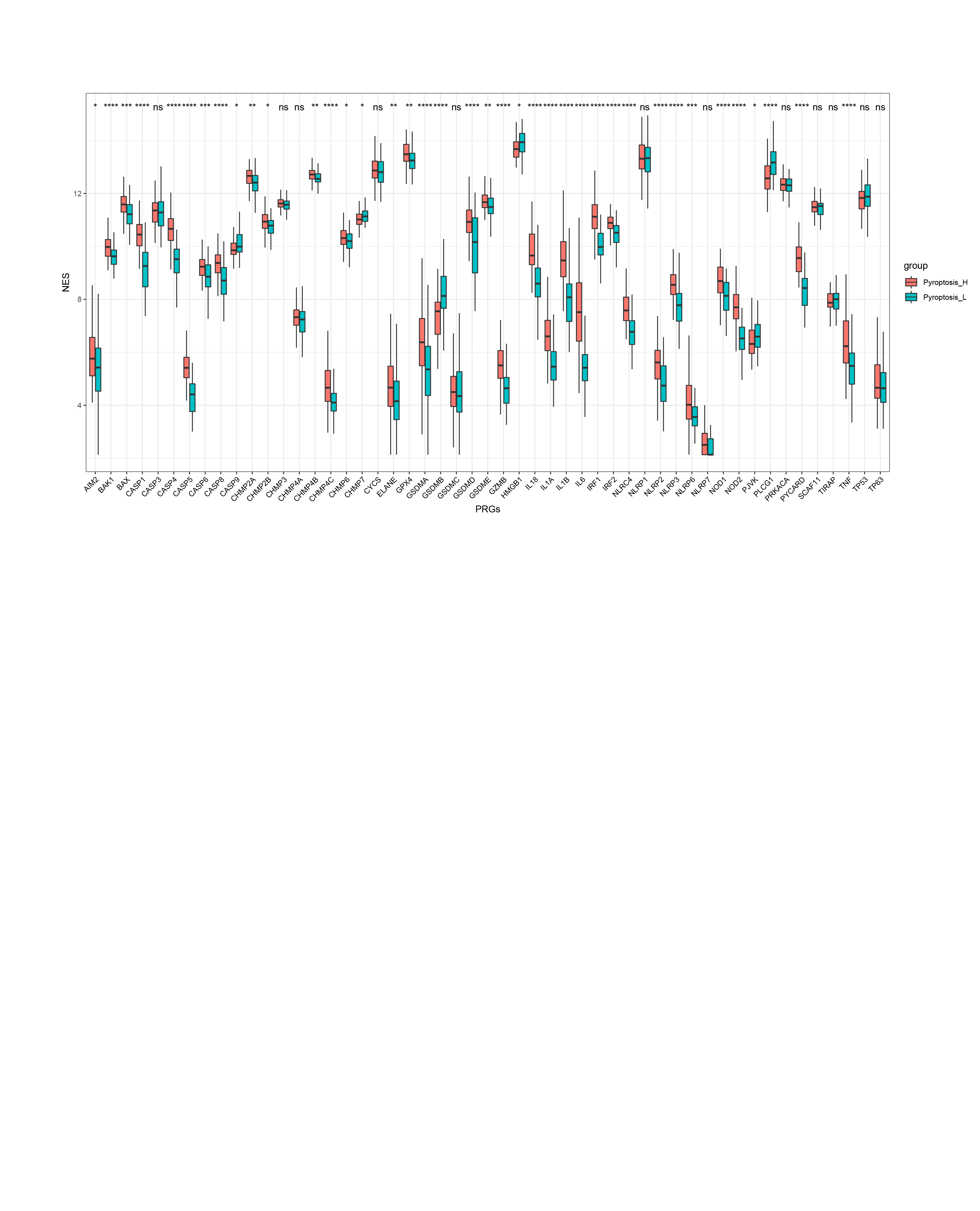

Supplement: Supplementary file 5 [file Image2.TIF]

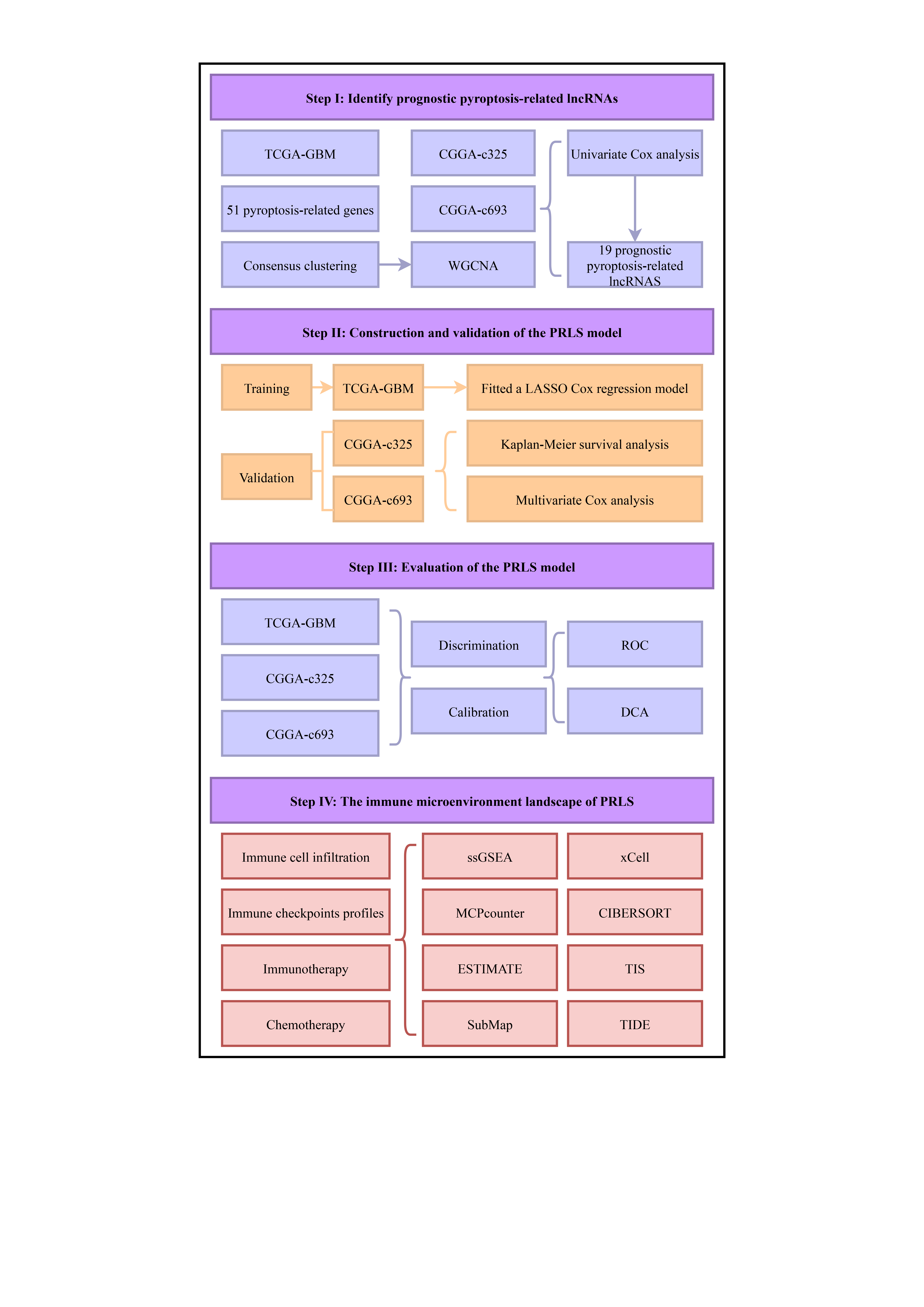

Supplement: Supplementary file 6 [file Image1.TIF]

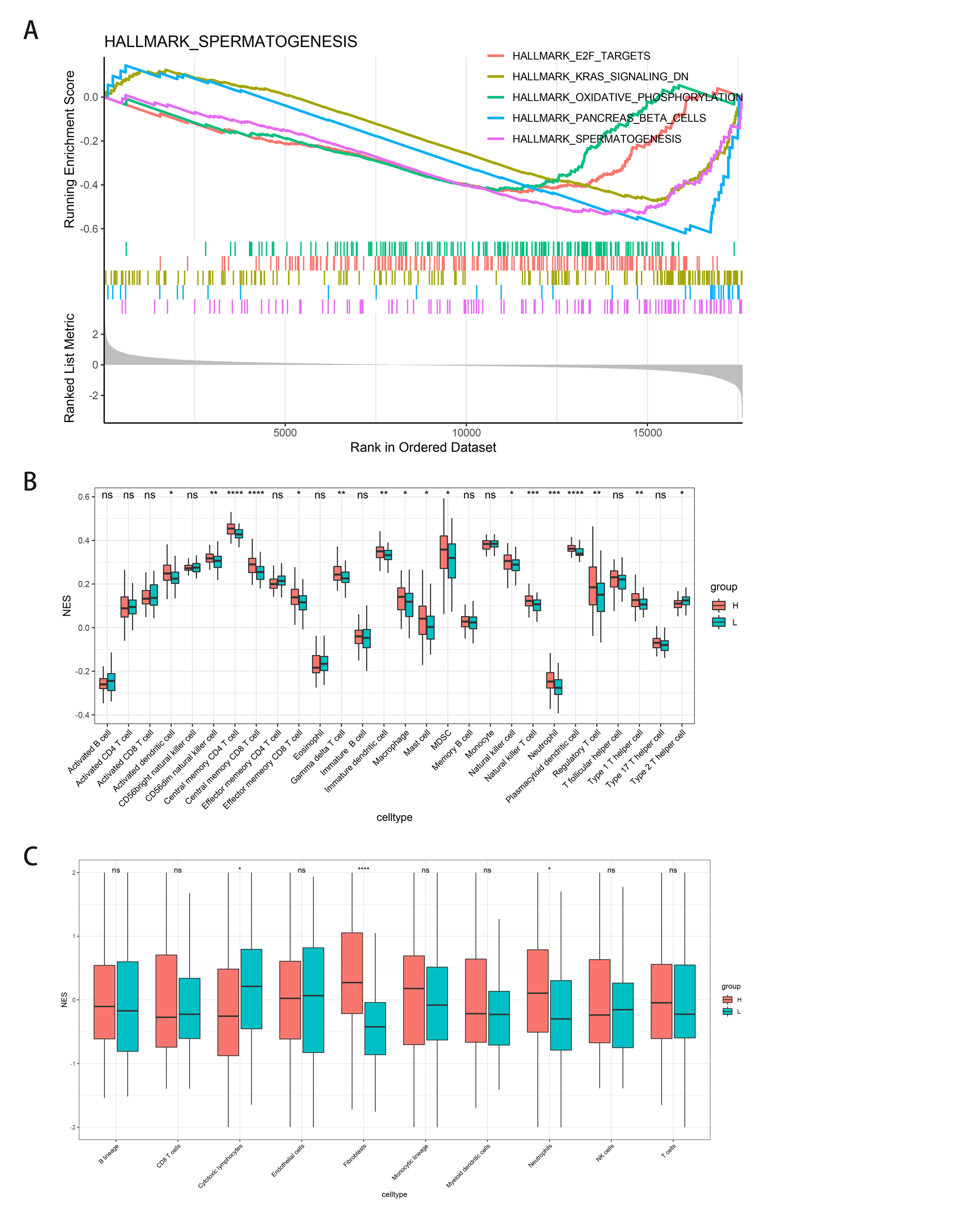

Supplement: Supplementary file 8 [file Image5.TIF]
